# Simultaneous Analysis of Multidrug Resistance 1(MDR1) C3435T, G2677T/A, and C1236T Genotypes in Hamadan City Population, West of Iran

**DOI:** 10.6091/ibj.1381.2014

**Published:** 2015-01

**Authors:** Massoud Saidijam, Hossein Mahjub, Nooshin Shabab, Reza Yadegarazari

**Affiliations:** 1 *Research Center for Molecular Medicine, Hamadan University of Medical Sciences, Hamadan, Iran;*; 2 *Dept. of Genetic and Molecular Medicine, Hamadan University of Medical Sciences, Hamadan, Iran;*; 3 *Dept. of Biostatistics and Epidemiology and Research Center for Health Sciences, School of Public Health, Hamadan University of Medical Sciences, Hamadan, Iran*

**Keywords:** Gene polymorphism, Multi-drug resistance, Neoplasm

## Abstract

**Background: **One of the limitations in the treatment of common diseases such as cancer chemotherapy is development of multidrug resistance (MDR). Polymorphisms could alter the expression level of *MDR1* gene, which plays an important role in MDR. In this research, the frequency of C3435T, C1236T, and G2677T/A polymorphisms of *MDR1* gene was investigated in a large group of population from Hamadan city to provide a sample data resource. **Methods:** Peripheral blood (2 ml) was taken, and DNA extraction was carried out. Multiplexed mutagenically separated PCR, which was followed by polyacrylamide gel electrophoresis and silver staining, was applied to detect the mentioned polymorphisms in 935 individuals. Sequencing performed for confirmation of gel electrophoresis resulted in 10 random cases. In total, alleles and genotypes of 933 persons (776 women and 157 men) were determined. **Results: **The most frequent alleles of the polymorphisms were: 3435T, C1236, and G2677. The most frequent genotypes were: 3435C/T, 1236C/T, and 2677G/A, and their concurrent presence was also found as the most frequent simultaneous genotypes. There was not any meaningful difference among the prevalence of these genotypes in groups of men and women. **Conclusion:** Our results were close to those of other studies performed in Iran and compared to the other ethnic groups, which showed more similarity to Asian peoples than Europeans. As an aspect of personalized medicine, it could be used by chemotherapists to improve the routine methods of cancer treatment.

## INTRODUCTION

The multidrug resistance 1 (MDR1) gene product, P-glycoprotein (P-gp), belongs to a family of ABC membrane transporters. This gene product is responsible for the cellular efflux of a variety of drugs and cellular metabolites across the plasma membrane and reduces exposure to potentially toxic compounds of intracellular environment [1-3]. Despite the advantage, this function results in resist-ance to a broad spectrum of drug effects, which in turn rises the side effects and costs of medical treatment of many important diseases in the field of general health e.g. cancers. The broad range of chemical transport affected by P-gp function consists of cancer chemotherapy and immuno-suppression agents and of drugs used in hypertension, allergy, infection, neuro-logy, and inflammation [4].

 The *MDR1* is a highly polymorphic gene, composed of 28 exons with a variety of single nucleotide polymorphisms (SNP) [5, 6]. Relationship between a few *MDR1* gene polymorphisms, presumably affects the P-gp function, and drug resistance has been studied in common malignancies, including lung [7, 8], breast [9, 10], colorectal [11], and gastric [12] cancers. Cancer risk may also be altered in the presence of these genetically predisposing factors [13]. Additionally, relationship between *MDR1* gene characteristics and gastric induced Helicobacter pylori infection [14] and also human immunodeficiency virus-1 infection [15] emphasizes the noteworthy function of *MDR1 *gene in all steps of prevention and treatment of diseases.

**Table 1:** Sequence and amounts of primers used for mutagenically separated PCR assays on multidrug resistance 1 (MDR1) gene three polymorphisms

**SNP alleles**		**F primer (amount of use** * **)**	**Common R primer** **(amount of use** * **)**	**Amplicon length (bp)**
G2677T/A	G	GAT AAG AAA GAA CTA GAG GGT G (1)	GAA AAA GAT TGC TTT GAG GAA TGG (5)	161
	A	GAC AAG ATC TGA AAT AAA AGA AAG AAC TAG TAG GTA (10)		175
	T	CAC TGA AAA TAA AGA AAG AAC TAG AAT GTT(10)		169
C3435T	C	GGT GTC ACA GGA AGA GAT C (1)	GGC CAG AGA GGC TGC CAC AT (1)	126
	T	CAG CCG GGT ATA GTC ACA GGA AGA TAT T (1)		135
C1236T	C	CCT GGT AGA TCT TGA ACG GC (2)	GCA TCA GCT GGA CTG TTG TG (20)	105
	T	CTC ACT CGT AAA GGT AGA TCT TGA AGA GT (20)		114

* , amount of use = picomol

C3435T polymorphism in exon 26 of *MDR1 *gene has a significant correlation with the P-gp expression level [16]. G2677T/A in exon 21 and C1236T in exon 12 are also other important polymorphisms of the gene. Bioavailability of many drugs may be influenced by these variants of *MDR1 *because of alteration in pharmacokinetics and pharmacodynamics of the drug [17]. However, functional significance of these polymorphisms of ABC membrane transporters has not been carefully understood; therefore, considering a particular SNP to predict individual's pharmacokinetics is not feasible.

Pattern of *MDR1 *polymorphisms differs among populations and races. Prediction of drug resistance and also an appropriate decision for medical treatment of many diseases are important results of studying these polymorphisms. Another aspect of importance of related studies is understanding the role of P-gp function in process such as apoptosis and immune responses [18]. Relationship of the polymorphisms of *MDR1 *gene and a few of diseases has been investigated in Iran [19, 20].

 In this investigation, we decided to determine the frequencies of C1236T, G2677T/A, and 3435T *MDR1 *gene alleles and genotypes in a large population from Hamadan city of Iran, which provides a primary data resource. These data help the clinicians to estimate the probability of vulnerability to many diseases and resistance to some of the important drugs, especially cancers and anti-cancer agents.

## MATERIALS AND METHODS

***Study group and sampling. ***The study group consisted of randomly chosen 935 recipients of primary health care, admitted to health centers of Hamadan city, Iran. The protocol was approved by Ethics Committee of Hamadan University of Medical Sciences prior to the study. The research was carried 

out according to the principles set out based on the Declaration of Helsinki 1964 and all subsequent revisions. After a brief explanation of study purpose and taking an informed consent, 2 ml peripheral venous blood was collected in sodium EDTA (as anti-coagulant)-containing tubes, kept on ice and transferred to laboratory. Then, the final results of the study on 933 individuals were reported.

***PCR analysis. *** DNA extraction kit (CinnaGen, Iran) was applied for DNA extraction according to the manufacturer’s instructions. The *MDR1 *SNP (C3435T, G2677T/A, and C1236T) profile was determined by a mutagenically separated PCR protocol. Allele-specific primers for each SNP were designed according to this protocol (Table 1). PCR amplification product lengths were different in various types of alleles [21] (Table 1). Advantages of the technique were as minimizing cross-reactions between primers and PCR products in ongoing cycles as well as simultaneous analysis of three genotypes of considered gene [22]. Primer efficacy was checked by preliminary tests on positive and negative controls. The reaction mixture contained 4 ng genomic DNA as template, 2 μM each dNTP, 1.5 mmol/l MgCl_2_, 0.5 unit of Taq polymerase, 3 µl PCR buffer 10×, and primers (as presented in Table 1). PCR cycles were: 1) early denaturation period of 10 min at 95°C, 2) 35 cycles of denaturation at 95°C for 45 s, annealing at 49°C for 45 s, and elongation at 72°C for 45 s, and 3) final extension at 72°C for 1 min.

***Gel electrophoresis. ***Detection of PCR products was carried out by subjecting the products to gel electrophoresis on pre-cast 20% TBE polyacrylamide gels at 90 V overnight and visualized by silver nitrate staining. Exposure to visible light and digital imaging was applied for revealing the amplicon bands and calculating their sizes compared to the standard ladder. Detection of bands with expected size (Table 1) was considered as positive result. 

**Fig. 1:**
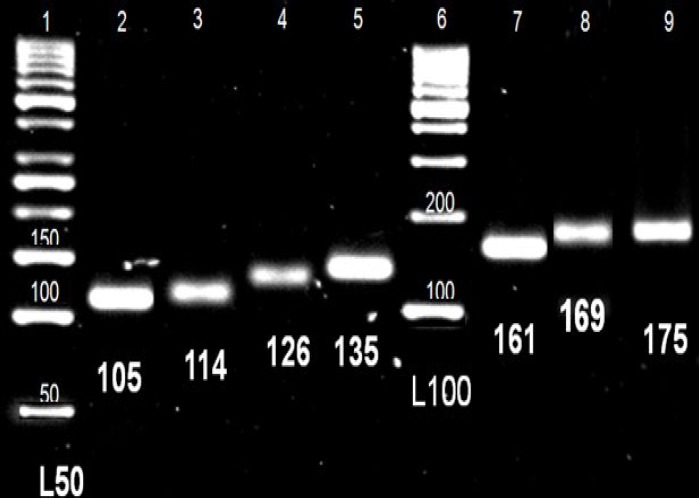
Polyacrylamide gel (20%) results after mutagenically separated PCR assays on multidrug resistance 1 (MDR1) gene C1236T, 3435T, and G2677T/A polymorphisms. Columns no. 1 and 6 show 50 bp and 100 bp ladder, respectively. Others show the detected bands of samples. The numbers indicate: allele C1236 (105 bp), allele 1236T (114 bp), allele C3435 (126 bp), allele 3435T (135 bp), allele G2677 (161 bp), allele 2677T (169 bp), allele 2677A (175 bp) and ladders bands.

***Sequencing*** ***analysis. ***Sequencing analysis was performed by Bioneer Corporation (Korea) on randomly chosen 10 PCR products.

***Statistical analysis. ***The sample size (n) was calculated based on results of allele and genotype frequencies for *MDR1 *SNP in similar studies using the following formula with prevalence (P) = 40% and difference (d) = 10%.The results were multiplied in 1.5 (as effect design) for calculation of final sample size. 

$n=\frac{{\left(1.96\right)}^{2}P(1-P)}{d^{2}}$

SPSS software (version 10) was used to calculate the frequencies of SNP. Two-sample binomial test was used to compare the results of frequencies. A *P *value <0.05 was considered as significant difference.

## RESULTS

Samples obtained from 933 individuals (157 males and 776 females) were analyzed. As mentioned in the previous section, in mutagenically separated PCR technique, making concurrent assay of several polymorphisms is possible. Figure 1 demonstrates an example of gel electrophoresis results. For any case, separate bands had been detected to reveal its polymorphism profile. 

 Results of allele and genotype profiling are presented in Table 2. Statistical analysis of the results showed that:

1. For SNP G2677T/A, allele G was the most frequent one and a meaningful difference was observed between the frequency of this allele and allele A (*P*=0.036) and T (*P*<0.001). In the case of genotypes, G2677A had a frequency more than the other studied genotypes (*P*<0.001).

2. For SNP C3435T, allele T showed a frequency more than allele C with no meaningful differences (*P*=0.293). In the case of genotypes, C3435T had a frequency more than the two other homozygous genotypes (*P*<0.001).

3. For SNP C1236T, allele C had a frequency more than allele T with meaningful differences (*P*<0.001). In the case of genotypes, C3435T had a frequency more than the other two genotypes (*P*<0.001).

For assessment of concurrent presence of *MDR1 *C3435T, G2677T/A, and C1236T polymorphisms, the most frequent genotype in each SNP group was considered, and the rates of their simultaneous presence were calculated (Table 3). As shown in Table 3, the most frequent case was concurrent presence of all of them.

Frequency of most common genotype in each group was also evaluated according to individuals’ gender. The parameter was statistically equal between men and women in all three genotypes (Table 4).

**Table 2:** Allele and genotype prevalence of multidrug resistance 1 (MDR1) gene polymorphisms in 933 individuals of Hamadan city population

**SNP**		**Allele frequency (%)**				**Genotype frequency (%)**					
G2677T/A		G 815 (43.7)	A 752 (40.3)	T 299 (16)		GG 150 (16.1)	GA 429 (46)	GT 86 (9.2)	AA 76 (8.1)	AT 171 (18.3)	TT 21 (2.3)
C3435T		C 917 (49.1)	T 949 (50.9)			CC 165 (17.7)	CT 587 (62.9)	TT 181 (19.4)			
C1236T		C 1192 (63.9)	T 674 (36.1)			CC 295 (31.6)	CT 602 (64.5)	TT 36 (3.9)			

**Fig. 2:**
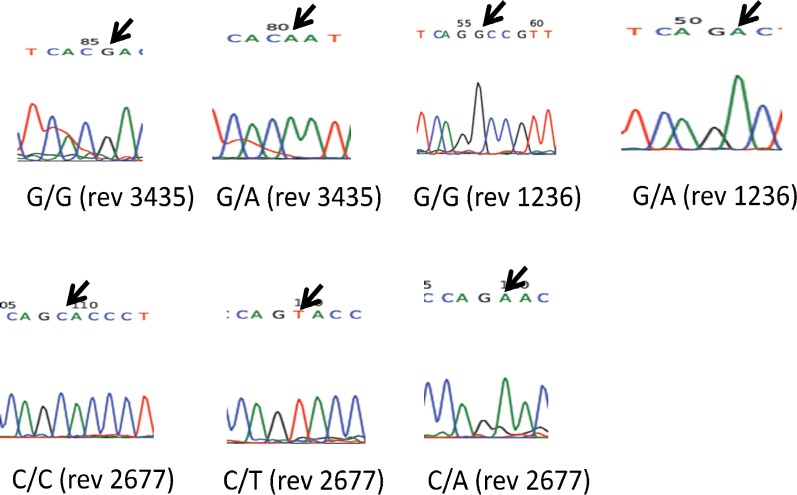
Sequencing results of mutagenically separated PCR products of multidrug resistance 1 (MDR1) gene C1236T, 3435T, and G2677T/A polymorphisms. rev, reverse sequencing

***Sequencing results.*** Sequencing analysis on 10 random chosen PCR products from different cases confirmed the accuracy of gel electrophoresis findings of PCR (Fig. 2). 

## DISCUSSION

Drug resistance is a common and vital challenge in the field of internal medicine. P-gp, the product of the *MDR1 *gene, plays an important role in the bioavailability of many medications with a narrow therapeutic window such as anti-cancer agents and cardiovascular drugs [22]. This phenomenon is the most common genetic mechanism of resistance to treatment with anti-cancer drugs. Many important data have been released from several investigations about the mechanism of P-gp function and pharmacokinetics of many commonly used drugs for circumventing this resistance and improving cancer chemotherapy [23]. Therefore, the function of human *MDR1 *gene may be Plus and minus signs indicate for individuals with or without studied genotypes, respectively. judged as a positive marker with ability to transforming human cells to MDR. Many hydrophobic anti-cancer agents have been shown the degrees of ineffectiveness due to this mechanism [24, 25]. Additionally, elevated levels of *MDR1 *RNA have been reported in many untreated cancers [26]. In some different neoplasms, the same condition has been described at relapse of the disease after chemotherapy [26].

**Table 3:** Concurrent presence of 3 most frequent genotypes of multidrug resistance 1 (MDR1) gene in 933 individuals of Hamadan city population

**Genotype**	**C3435T +** **C1236T -**	**C3435T -** **C1236T +**	**C3435T +** **C1236T +**	**C3435T -** **C1236T -**
G2677A +	92	96	196	45
G2677A -	117	128	182	77

More than one hundred SNP have been recognized in the human *MDR1 *gene [27]. The most important are C3435T, C1236T, and G2677A, T SNP. In the present study, we decided to determine the prevalence of these functionally important SNP in a large population to provide valuable information for optimizing the individualized therapeutic approach especially for anti-cancer treatment.

In the case of C3435T, the most studied poly-morphism in cancers, the frequency of heterozygote genotype (C3435T) was more than two other genotypes in both male and female subjects. The same results have been also released from similar recent studies in Iran [19, 28-30]. Result of a study in Japan on 154 healthy Japanese and 100 healthy Caucasians showed the same result for genotypes in Japanese but a more frequency of genotype T3435T in Caucasians [31] and this study. The frequency of genotype T3435T has been also shown to be higher in Germany and Serbian ethnics [22, 32]. In total, it seems that the pattern of SNP of C3435T polymorphism in Iran resembles to Asian peoples in comparison to Europeans. The expression of* MDR1 *gene and the probability of drug resistance have been shown to be lower in carriers of genotype T3435T than C3435C [21, 33]. However, recent meta-analysis studies have affirmed that presence of T3435T genotype correlates with increase in the risk of colorectal cancer and acute lymphoblastic leukemia [13, 34]. 

**Table 4:** Comparison of prevalence of 3 most frequent genotypes of multidrug resistance 1 (MDR1) gene between men and women in 933 individuals of Hamadan city population

** Genotype**	**2677G/A (%)**	**3435C/T (%)**	**1236C/T (%)**
**Sex**			
males	82 (52.2)	98 (62.4)	112 (71.3)
females	347 (44.7)	489 (63)	490 (63.1)
*P* value	0.085	0.888	0.051

Allele G2677 and genotype G2677A were more prevalent in G2677A/T polymorphism than the others. Results for the mentioned allele were similar to Japanese, Caucasians, and Serbians with small differences, but genotype frequencies, in this study, had a significant difference with the last two mentioned groups [22, 31]. Unexpectedly, similar investigations in Iran showed dissimilar findings [28, 30]. Overall risk of cancers increases in individuals with TT, AT, and AA genotypes, especially Asian populations [34]. 

In C1236T group, genotype C1236T was more common than the other genotypes in our study, which was similar to Japanese, Caucasians, and Serbians [22, 31]. However, the more frequency of allele C than T is different from the results of many other studies but for Serbian people. Not so many information has been released regarding to the prevalence of C1236T genotype, and also no correlation between this genotype and cancer has been found [34].

Function of membrane transporter such as *MDR1 *was affected by special mutations, which may be considered as a predictive marker in both the risk of diseases and their mortality and morbidity [16-19]. Therefore, the evaluation of the prevalence of these SNP in the present study and ongoing researches in other countries and populations would induce new concepts about investigation in the field of personalized medicine and could be applied as a primary data for this purpose. Additionally, the participants of such studies may be the subjects of future ante-grade cohort researches for assessment of risk ratios of many important diseases.
